# Family-based improvement for health literacy among the Yi nationality (FAMILY) in Liangshan: protocol of an open cohort stepped wedge cluster randomized controlled trial

**DOI:** 10.1186/s12889-022-13782-w

**Published:** 2022-08-13

**Authors:** Lin Hu, Wenhui Zhu, Jie Yu, Ying Chen, Jingmin Yan, Qiang Liao, Tao Zhang

**Affiliations:** 1 Department of Epidemiology and Health Statistics, West China School of Public Health and West China Fourth Hospital, Sichuan University, Renmin South Road 3rd Section NO.16, Chengdu, 610041 Sichuan Province China; 2 Liangshan Prefecture Center for Disease Control and Prevention, Xichang, 615000 Sichuan Province China

**Keywords:** Health literacy, Family branch system, Contracted family doctor services, Stepped wedge cluster randomized trial, Mixed-effects model, Complier average causal effects

## Abstract

**Background:**

Improvement of health literacy constitutes a cornerstone to improving public health. However, the overall health literacy of Liangshan Yi Autonomous Prefecture (Liangshan Prefecture) in the southwest Sichuan Province of China has kept extremely low for a long time. How to improve health literacy of the Yi nationality residents is key to be urgently solved. Notably, Family Branch System is a distinctive patrilineal bloodline organization of Yi nationality, which plays an important role in the daily life of Yi nationality. Meanwhile, Contracted Family Doctor Services is conducted in Liangshan Prefecture. Therefore, this study proposes an intervention model of health education based on Family Branch System and Contracted Family Doctor Services, which is a Family-based Improvement for Health Literacy among the Yi nationality (FAMILY) in Liangshan, when improving traditional Innovative Care for Chronic Conditions Framework (ICCC) framework.

**Methods:**

An open cohort stepped wedge cluster randomized trial design is used to implement health literacy education interventions including project preparation, core group building, promotion within family branch and competition between family branches while using Contracted Family Doctor Services as control measure. The study will be conducted among Yi nationality residents in Meigu County and Yanyuan County, with health literacy level of residents as the primary outcome. Finally, mixed-effects model and causal inference method will be used to evaluate intervention effect.

**Discussion:**

This study highlights family, using the unique Family Branch System and Contracted Family Doctor Services in Liangshan Prefecture to design intervention among improved ICCC framework, and combines the mixed-effects model with complier average causal effects (CACE) to estimate the intervention effect under non-compliance for the first time. Besides, other key technologies to be adopted include construction of electronic questionnaire quality control system, with quality control based on artificial intelligence. This trial contributes to exploring an effective way to improve health literacy of Yi nationality residents in Liangshan Prefecture, which will provide reference for other areas, especially poor areas, to improve residents’ health literacy.

**Trial registration:**

ISRCTN11299863 on June 1, 2022; https://www.isrctn.com/.

**Supplementary Information:**

The online version contains supplementary material available at 10.1186/s12889-022-13782-w.

## Background

Improvement of health literacy constitutes a cornerstone to improving public health [1, 2]. It is an important strategy and measure to improve people’s health, an important part of promoting constructing Healthy China, and one of the main indicators in the *Outline of the Healthy China 2030 Plan* [3]. However, under influence of economic development and historical changes, the health literacy level of residents in Liangshan Yi Autonomous Prefecture (Liangshan Prefecture), where located in the southwest Sichuan Province of China, is still at a low level (only 11.62%). This level is lower than that of Sichuan province in 2019 (19.40%) and rural residents in 2017 (14.5%), indicating a big gap between the overall requirement of the *Outline of the Healthy China 2030 Plan* [3] still exists. In addition, the health literacy level of residents in some Yi autonomous counties are even only about 1% [4]. Therefore, how to improve the health literacy of the Yi nationality residents is a key issue to be urgently solved in the realization of *the Outline of the Healthy China 2030 Plan* in Liangshan Prefecture.

International scholars have conducted in-depth research on how to improve the prevention and management of Noncommunicable diseases (NCDs). Among many proposed models and theories, Chronic Care Model (CCM) and Innovative Care for Chronic Conditions Framework (ICCC) have been widely recognized and applied [5]. Compared with CCM, ICCC framework is more specific, operational, and suitable for low- and middle- income regions. The ICCC framework elucidates that individuals and their families are an undervalued asset in health care system, which emphasizes the role of individuals and families, and the need to promote them to partner with communities and health care organizations [6, 7]. Positive outcomes in NCDs management can only be achieved when individuals, family, community partners and healthcare working groups are informed, motivated, prepared and work together [8].

As mentioned above, family could make an important contribution to health promotion. Thus, in the process of improving the Yi nationality residents’ health literacy in Liangshan Prefecture, we should fully respect the local ethnic customs and habits, and also give full play to the role of family, which could be achieved through Family Branch System and Contracted Family Doctor Services. Yi nationality is a mountainous nation, many of whom live in the mountains with relatively backward traffic, making them have little contact with the outside and less influenced by foreign culture [9, 10]. Its traditional culture has a greater influence on the social life and play an important role in the process of social development. Notably, Family Branch System is a distinctive patrilineal bloodline organization of Yi nationality in ancient times [11], which has been passed down to this day and plays a vital part in the daily life of Yi nationality. A large number of practical experiences show that during the transition from slave society to socialist society in Liangshan Prefecture, Family Branch System has exerted a significant influence on drug control, grass-roots governance and legal construction [12]. For example, at the end of 2017, the National Health Council and Sichuan Provincial People’s Government jointly launched and implemented the AIDS prevention, treatment and health poverty alleviation campaign in Liangshan Prefecture [13]. In the special implementation process of management capacity building, Liangshan Prefecture has carried out pilot work in its Meigu County and Yanyuan County in response to the problems exposed in grass-roots PMTCT management [14], expanded the scope of capacity building from business and service support to comprehensive management, and fully utilized the key personage of the family branch. In addition, many foreign studies also showed that in less developed countries and regions such as sub-Saharan Africa, tribal culture similar to the family branch system was also critical in environmental protection and HIV prevention and control [15, 16]. Meanwhile, Contracted Family Doctor Services [17, 18] is conducted throughout the state in accordance with the unified requirements of the National Health Commission in Liangshan Prefecture. An important part of this work is to build an interactive platform for family doctors and contracted residents, providing services such as appointments, consultation, health management, and follow-up visits for NCDs. Regularly and accurately push health education information according to different service needs, seasonal characteristics and epidemic situation [19, 20]. Therefore, taking advantage of the Family Branch System and Contracted Family Doctor Services in Liangshan Prefecture is expected to be key in improving the residents’ health literacy.

To this end, this study intends to learn from the experience of AIDS management in Liangshan Prefecture, and proposes an intervention model of health education based on Family Branch System and Contracted Family Doctor Services, which is a Family-based Improvement for Health Literacy among the Yi nationality (FAMILY) in Liangshan, when improving the traditional ICCC framework by strengthen the connection of the three elements at micro level.

Finally, to evaluate the effect of intervention measures while achieving full coverage of beneficial measures, we will adopt stepped wedge cluster randomized trial (SW-CRT), which is widely used in health education, medical staff training and other trial with interventions that benefits outweigh the disadvantages [21–24]. SW-CRT usually has no special control groups, and as the trial goes on, each group will accept intervention sequentially by random, alleviating ethical and moral problems to a large extent when saving resources [25]. Besides, due to the large local population prevalence in Liangshan Prefecture such as migrant workers, etc., some of the study subjects may be lost to follow-up with others may join in the middle process of the study. In view of the above, an open cohort SW-CRT is used in this study [26], with specific study design showed below.

## Methods

### Aims

#### Primary study aim

Propose a set of health literacy intervention mode for the Yi nationality residents in Liangshan Prefecture to improve the health literacy, based on the family.

#### Secondary study aim

Improve the statistical analysis method of SW-CRT, and propose a hybrid model-based main hierarchical causal inference method to solve the common non-compliance problems in SW-CRT.

### Design

This manuscript adheres to the Extensions of the CONSORT Statement and SPIRIT Statement (Additional files 1 and 2).

#### Open cohort stepped wedge cluster randomized trial design

**Fig. 1:**
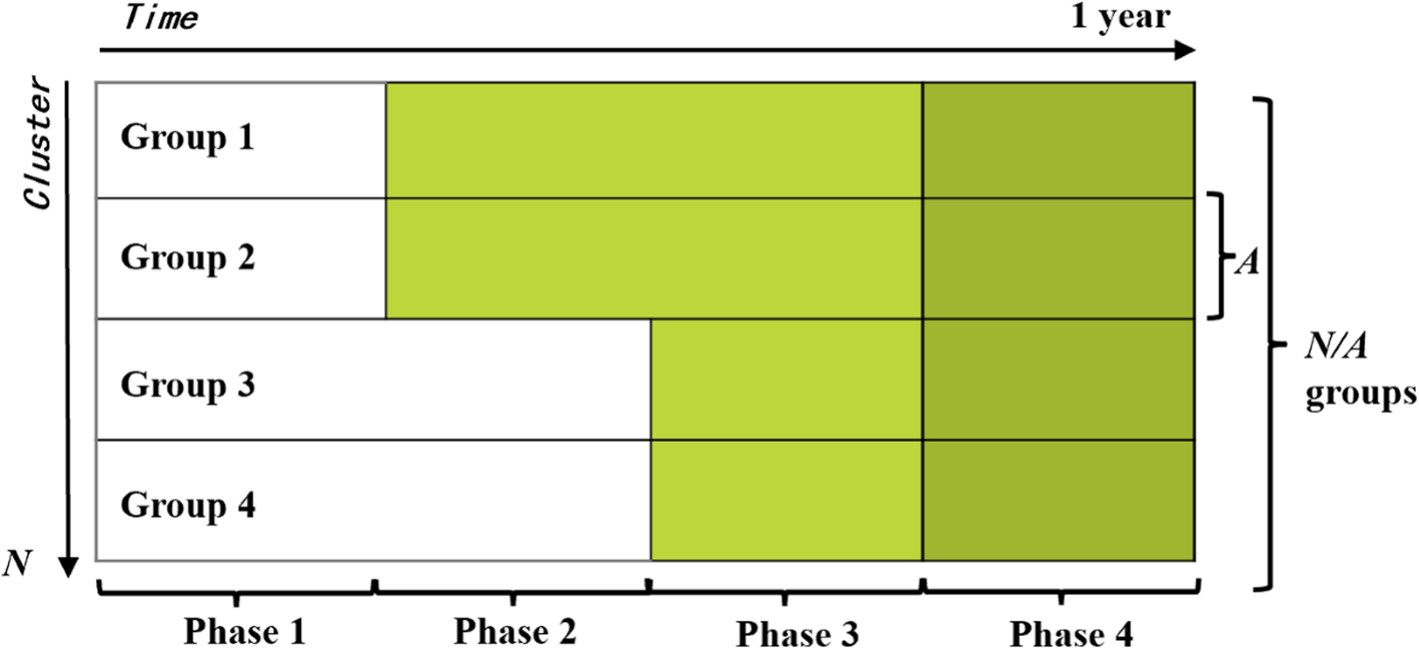
Stepped wedge study design

For this study, *N* family branches in the Meigu and Yanyuan County will be included. Each family branch is regarded as a cluster, and *A* clusters constitute a group. It is assumed that there are *N* / *A* groups in the study, the first and third groups are in Meigu County and the second and fourth groups are in Yanyuan County. The experimental study period of the whole project is 1 year, from May 2022 to May 2023. All groups are in non-intervention during the first 3 months (Phase1) of the project. In the second 3 months (Phase2), groups 1 and 2 will start to receive intervention. Then 3 months later (Phase3), groups 3 and 4 will begin to receive intervention. According to the research proposal, all groups will continue to increase 3-month follow-up (Phase4), after all of them having received intervention in a random order, ensuring that study outcomes can be observed when time lag effect of intervention exists (Fig. 1).

#### Study site

The trial is set in Meigu County and Yanyuan County, Liangshan Prefecture, Sichuan Province, China, where have complex and changeable landforms [27]. Meigu and Yanyuan are far apart in geographical location. Specifically, many villages are still dominated by winding mountain highway, and some villages even have no pass roads. The family branches are in a relatively closed state with less communication, which could be helpful to prevent the contamination between the intervention and non-intervention family branches. In addition to the geographical advantage, Meigu and Yanyuan County have also carried AIDS management work, providing practical conditions for the experiment [28, 29].

#### Study object

The project is based on the community health service centres and related medical institutions [30] in the selected districts. Residents will be included if they met all conditions: (1) 15–64 years old, (2) Yi nationality residents living in compact communities, (3) have been living in the district for more than 6 months, (4) native place is Liangshan Prefecture, (5) voluntarily participate in the project and willing to provide informed consent.

People who do not agree to participate and who are unable to participate (such as minors, dementia, schizophrenia and so on) will be excluded.

#### Intervention

**Fig. 2:**
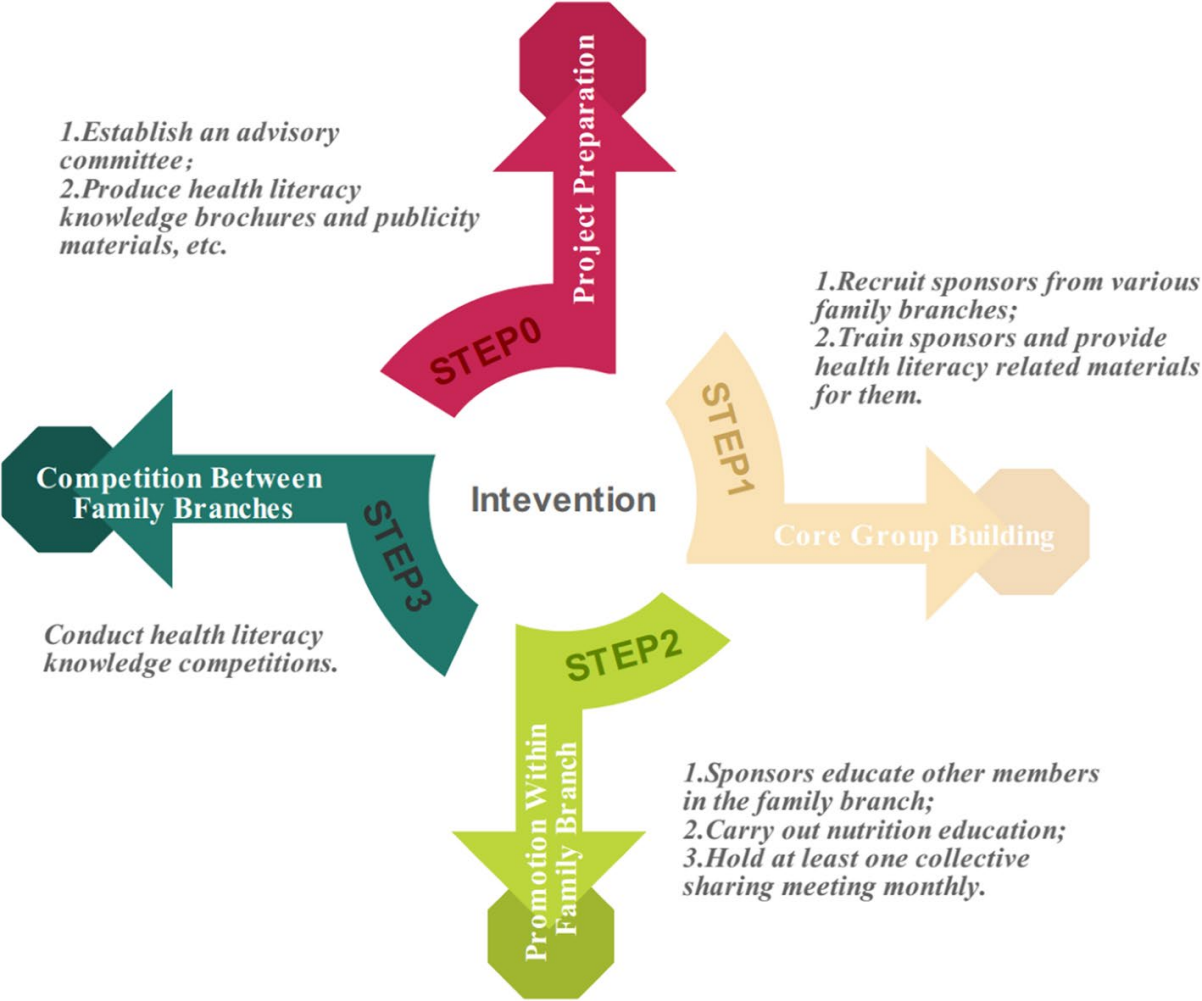
Intervention strategy

The intervention is health literacy education, including project preparation, core group building, promotion within family branch and competition between family branches (Fig. 2), and details are as follows.

##### Step 0. Project preparation

Organize experts to prepare health literacy knowledge brochures and make operating manuals and activity calendars with reference to the *66 Articles of Health Literacy for Chinese Citizens* [31, 32]. The experts include the experts of health education and NCDs prevention and control from Sichuan University, the experts from Sichuan Center for Disease Control and Prevention (CDC), the head of Health Education Institute and the hard-core of Chronic Infectious Disease Prevention and Control Institute in Liangshan CDC, the director of Health Committee of Liangshan, an expert in the Communication and Health Promotion Department and an expert in Disease Prevention and Control Department in Health Committee of Liangshan, the directors and deputy directors of Meigu CDC and Yanyuan CDC, and an expert from Xichang Community Health Service Center. According to the health literacy knowledge brochures, we produce publicity materials such as towels, umbrellas, and cell phone charging cables based on health knowledge.

The above experts are organized to establish an advisory committee to provide supports including health knowledge, health skills, information technology, and financial to each family branch sponsor, etc.

##### Step 1. Core group building

The sponsors from various branches are recruited by township hospitals, and written and interview examinations are conducted by the advisory committees. This project will train sponsors, including health literacy related knowledge and group promotion training. Moreover, we will provide the sponsors with operating manuals, activity calendars, publicity materials, health literacy knowledge brochures, etc.

##### Step 2. Promotion within family branch

The sponsor of each famliy branch will use the health literacy knowledge brochures to educate other members in famliy branches on health literacy according to the operating manual. We encourage all members of famliy branches to participate in the project, identify problems, propose and implement solutions, conduct self-evaluation and measure improvement. Besides, we require each famliy branch to hold at least one collective sharing meeting monthly.

In terms of nutrition education, we will design and produce nutrition education playing cards in the form of popular 4 or 6 sentences, which are repeatedly revised and proven by a number of nutrition experts. The contents mainly include Chinese dietary guidelines and balanced diet pagoda, the function of nutrients and their main food sources, the nutritional value of various foods, dietary guidance for specific populations and patients with common NCDs, aerobic exercise, weight evaluation and its impact on health, food safety problems, the harm of excessive smoking and drinking, etc. The nutrition education methods will include the following: (1) Distribute nutrition education cards, introducing their main contents and carrying out a quiz on the contents of the cards. Train the sponsors to explain the cards to other family members through their publicity. (2) Distribute nutrition health knowledge publicity materials, and ask the use of the cards to supervise. (3) Set up nutrition knowledge bulletin boards and post nutrition health knowledge bulletin materials. (4) Start nutrition knowledge courses and lectures. (5) Set up personalized online or offline nutrition counseling and guidance stations which are mainly based on the actual dietary situation of the residents, and provide relevant dietary nutrition guidance after analysis.

We will organize training and seminars for the members of the advisory committee, the heads of project health center, and the sponsors of each family branch. In the meetings, each family branch sponsor needs to give feedback on the problems encountered in the intervention implementation process, and the committee members will make suggestions in response to the problems. We will train the sponsors on health literacy knowledge and distribute the publicity materials. Besides, the family branches are encouraged to exchange learning experiences. Regularly invite leaders of the Liangshan Prefecture Health Committee to attend the meeting to report on the interventions in the recent months, and receive advice and guidance.

##### Step 3 competition between family branches

Take advantage of traditional Yi festivals to conduct health literacy knowledge competitions on a regular basis by family branches. We will distribute the prizes according to the competition rankings, and issue the honorary certificates to the family branches with outstanding scores. Use the concept of family honor of Yi nationality to stimulate the internal motivation of residents to improve health literacy, exam and test the actual mastery of health literacy knowledge through competitions and other means.

#### Control

The project uses Contracted Family Doctor Services as a control measure, which is currently an important routine measure to improve residents’ health literacy in Liangshan Prefecture. Therefore, the project takes it as a control measure and compares it with the effect of intervention measures.

#### Outcome

The primary outcome of the project is the health literacy level of residents in the *Health Liangshan Yi Autonomous Prefecture Performance Evaluation Index*, which is defined by the 2020 *Residents Health Literacy Monitoring Questionnaire* [33].

The secondary outcomes are prevalence of hypertension, prevalence of diabetes, BMI, smoking intensity index [34], and alcohol consumption [35]. The project will measure the outcome indicators at 1, 3, 6, 9, and 12 months.

### Data collection

**Fig. 3:**
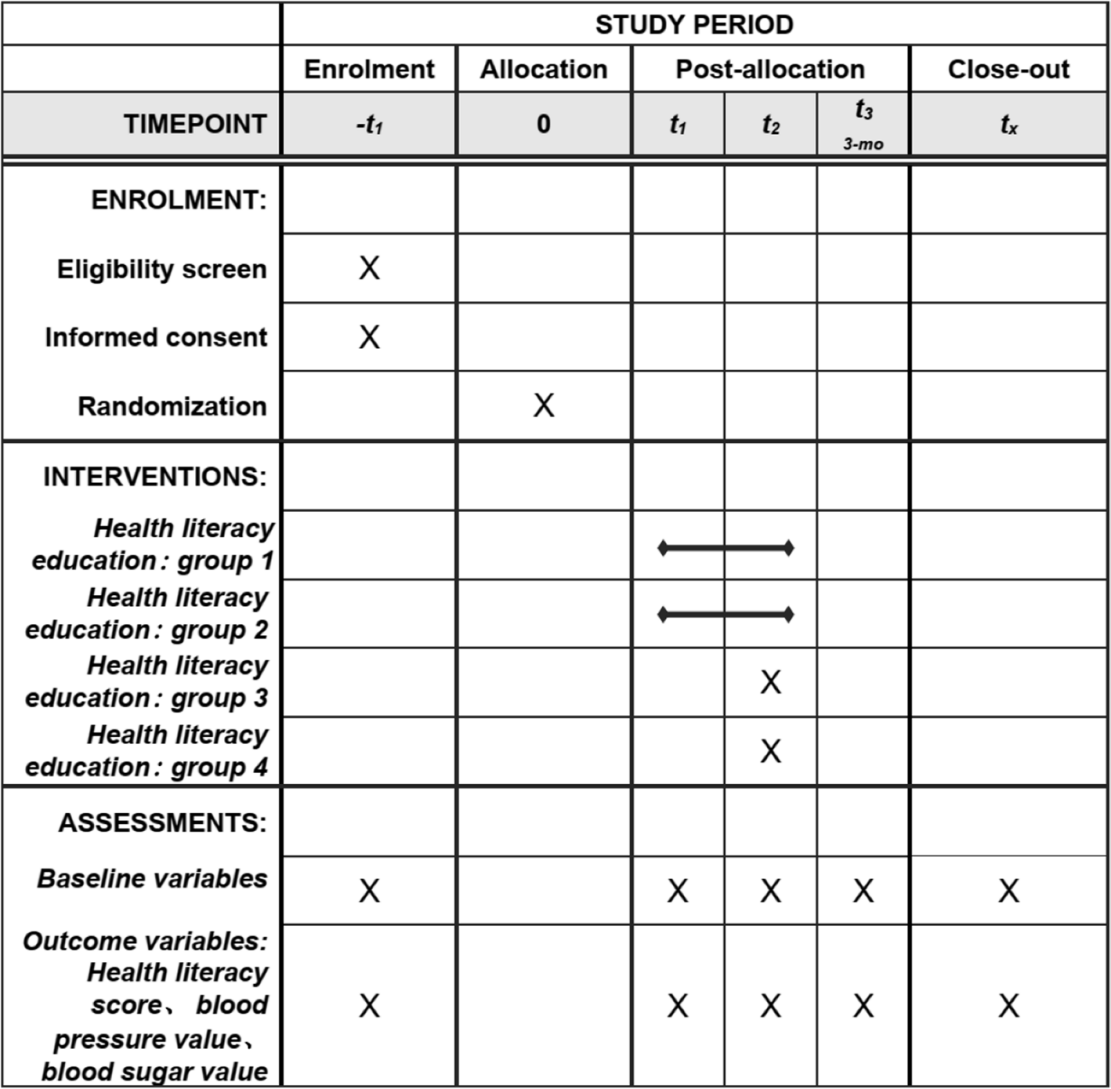
SPIRT Figure

Each township health center is responsible for the recruitment and training of data collectors. The data collector will help participants complete a questionnaire and collect physical examination information at the 1, 3, 6, 9, and 12 months (Fig. 3). This study uses the 2020 National Residents’ Health Literacy Monitoring Questionnaire, which includes three basic aspects and six categories of questions about health literacy [33]. Points are scored according to questions related to the questionnaire, meaning 1 point for correct answers to single-choice questions and 0 point for incorrect answers, 2 points for correct answers to multiple-choice questions and 0 points for incorrect answers (less choice, more choices, and wrong choice are all judged as wrong answers). The total score of the questionnaire is 66 points.

Data collectors will also collect information on smoking and alcohol consumption, and measure participants’ height, weight, blood pressure, and blood sugar. The sponsor of family branch will inform the participants of the time and place of measurement, and the need to fast for one night. All participants will be informed of the study information orally and in writing, prior to the questionnaire and physical examination, and their data will be collected only after providing written informed consent.

Data collection will follow standard data management protocols. The research will provide suitable survey sites, and assign special personnel to be responsible for on-site supervision, double data entry, data coding, data verification, and data preservation. Only members of the research team have access to the data, and identifiable data will not be collected and will be replaced by serial numbers to reduce the risk of privacy disclosure, so there will be no risk to residents’ privacy.

### Sample size and sampling method

This study is an open cohort design. Since the withdrawal of the original cohort participants and the addition of new participants during the progress of the longitudinal study are allowed, the number of trial measurements may be different for each participant. Hence, after discussion with local experts, this study intends to set the participation retention rate to 0.6.

Use the formula for calculating the sample size of an open cohort SW-CRT proposed by Kasza et al. in 2020 [36], assuming that there are 50 subjects in each cluster-period, the intra-cluster correlation coefficient is 0.4, the cluster autocorrelation coefficient is 0.9, the individual autocorrelation coefficient is 0.7, the total variance is 19, and decays in between-period correlations and participant-level correlations are allowed. On the significance level α = 0.05, the 1 difference of outcome effect after intervention would be found. At the same time, in order to ensure 80% power, the minimum number of clusters required is 7.699, rounded up to 8, making the total test subjects are 400 cases. In order to take non-response into account, we expanded the sample size by 30%, and obtained 572 subjects and 11.44 clusters. After rounding the number of clusters, finally, 600 subjects from 12 family branches will participate in the study. Using R 4.0.5 to generate random numbers and assign a unique number to each recruited family branch in Meigu County and Yanyuan County. Simple random sampling will be used to select 6 family branches from Meigu County and Yanyuan County respectively, and completely randomized into 4 groups independently. In the next stage, a random sample of 50 eligible individuals will be stratified by gender and age within each family branch. The generation of random allocation sequence and allocation are completed by a third-party independent statistician.

The Meigu County and Yanyuan County carry out various health education activities for residents’ health literacy annually. This study will enrich the health literacy improvement plan under local routine management work. Due to the low education level and relatively occluded information in the study sites, it is difficult for subjects to perceive transition from the non-intervention state to the intervention state, ensuring implementation of blinding for the subjects to some extent.

### Statistical analysis plan

According to the design of this study, the mixed-effects model will be used to estimate the intervention effect, combined with the causal inference model to deal with non-compliance issues.

#### Establish mixed-effects model to evaluate intervention effect

We will establish a mixed-effects model for open cohorts. To account for the inter-stage and intra-individual correlation decay, we would use the blended correlation decay model for open cohorts proposed by Kasza et al., and use the generalized least squares estimation to estimate the intervention effect [37]. The mixed-effects model is as follows:

1 $$\left\{\begin{array}{c}{Y}_{ij k}=\mu +{\beta}_j+\delta {X}_{ij}+{\gamma}_{ij}+{\varepsilon}_{ij k}\\ {}{\gamma}_i={\left({\gamma}_{i1},\cdots, {\gamma}_{iJ}\right)}^{\hbox{'}}\sim N\left(0,{\tau}_{\gamma}^2\boldsymbol{M}\left(1,r\right)\right)\\ {}{\varepsilon}_{ik}={\left({\varepsilon}_{i1k},\cdots, {\varepsilon}_{i{J}_kk}\right)}^{\hbox{'}}\sim N\left(0,{\sigma}_{\varepsilon}^2\boldsymbol{M}\left(1,\eta \right)\right),{\boldsymbol{\varepsilon}}_{ik}\perp {\boldsymbol{\varepsilon}}_{im},k\ne m\\ {}\boldsymbol{M}\left({r}_0,r\right)=\left(\begin{array}{ccccc}1& {r}_0r& {r}_0{r}^2& \cdots & {r}_0{r}^{J-1}\\ {}{r}_0r& 1& {r}_0r& \cdots & {r}_0{r}^{J-2}\\ {}\vdots & \vdots & \vdots & \ddots & \vdots \\ {}{r}_0{r}^{J-1}& {r}_0{r}^{J-2}& {r}_0{r}^{J-3}& \dots & 1\end{array}\right)\end{array}\right..$$

Where *i* represents the *i* − *th* group; *j* represents the *j* − *th* stage; *k* represents the *k* − *th* group; *μ* is the grand mean; *β*_*j*_ is a fixed effect of stage *j* used to correct time trends; *δ* is the fixed effect of intervention; *X*_*ij*_ is the time-varying intervention indicator of group *i* in stage *j*; *γ*_*ij*_ is the random intercept of group *i* in stage *j*; *ε*_*ijk*_ is the individual specific random error; ***M*** is a structure matrix including two parameters; *r* and *η* are the decay rates at the cluster-period level and individual level, respectively.

#### Adopt causal inference model to deal with non-compliance issues

SW-CRT can observe participants’ compliance under intervention and control conditions [38, 39]. Therefore, we would divide the participants into four subgroups based on their compliance, as shown in Table 1. *D*_*i*_(0) and *D*_*i*_(1) represent that individual *i* is divided into control group and treatment group, respectively. *D*_*i*_ = 0 and *D*_*i*_ = 1 represent individual *i* actually accept control and actually accept treatment, respectively.

**Table 1:** Treatment Usage by Subgroup Classification

Behavior	Subgroup
*D*_*i*_(0) = 0, *D*_*i*_(1) = 1	Complier
*D*_*i*_(0) = 1, *D*_*i*_(1) = 1	Always-taker
*D*_*i*_(0) = 1, *D*_*i*_(1) = 0	Defier
*D*_*i*_(0) = 0, *D*_*i*_(1) = 0	Never-taker

Below, lay out the steps we followed to identify and estimate complier average causal effects (CACE) in our SW-CRT.

##### Treatment of interest

In this study, we attempt to identify the effect of compliance with Yi nationality residents in Liangshan Prefecture through the health literacy intervention based on Family Branch System.

##### Causal model of compliance & classification of subgroups

In our study, the control group can be treated, so we construct subgroup classifications based on cumulative behaviors to account for time-varying treatment-usage patterns. We will divide participants into four subgroups, including Complier, Always-taker, Defier, and Never-taker.

##### Equivalence of comparison groups

To check for balance in observable characteristics among compliers, calculate a weighted average of baseline characteristics by the amount of time a Family branch spent in either control or intervention time periods. For example, if a Family branch crossed over in step *a*(0 < *a* < *n*), it contributed baseline characteristics to *a* control periods (step 0~*a* − 1) and *n* − *a* + 1 intervention periods (step *a*~*n*).

##### CACE estimation

We will estimate inter-group differences in time-dependent SW-CRT for outcomes:

2 $$E\left[Y|A=1,T=t,C=1\right]-E\left[Y|A=0,T=t,C=1\right],$$

where *Y* is the outcome; *A* is an indicator, *A* = 1 is the treatment group, *A* = 0 is the control group; *T* represents time; *C* is an indicator of being a complier (*C* = 1). Under the above assumptions, we supposed *C* is a preexisting characteristic and is stable throughout the study period. The estimation of step-specific CACE can be averaged over all steps to avoid the impact of time and interventions.

We assume that the intervention prior to step *t* does not affect outcomes at step *t* and the intervention effects are constant (*E*[*Y*| *A* = 1, *T* = *t*, *C* = 1] − *E*[*Y*| *A* = 0, *T* = *t*, *C* = 1] is not a function of *t*). Then the estimation of step-specific can be averaged of the effect across all intervention steps:

3 $$\frac{1}{n}\sum \limits_{i=1}^n\frac{1}{s}\sum \limits_{t=0}^sE\left[{Y}_i|{A}_i=1,{T}_i=t,C=1\right]-E\left[{Y}_i|{A}_i=0,{T}_i=t,C=1\right],$$

where *n* is the total number of participants; *s* is the total number of steps.

The behaviors of participants were observable in SW-CRT. So, we can estimate CACE according to mixed-effects model based on the outcomes of complier. As shown in formula (1), *δ* is CACE.

### Quality control

In order to ensure the validity and accuracy of study data, we comply with the national model queue technical standards strictly. In the preparation stage, we will combine with the natural geography and national characteristics of Liangshan Prefecture to formulate standard operating procedures (investigation process and norms, questionnaire survey instructions and training manuals, etc.) for the whole process of the survey, and conduct pre-survey to verify the validity and accuracy of the survey plan and questionnaire. We recruit investigators who can speak dialect, have medical knowledge and experience in epidemiological investigations, and then conduct unified training. We also combine community publicity to improve the enthusiasm and cooperation of the masses.

In addition to the above traditional quality control measures, we will build an electronic questionnaire quality control system and a face-to-face recording survey process. In this system, the quality control algorithm based on artificial intelligence and the outlier detection algorithm based on the minimum covariance determinant (MCD) are embedded [40, 41], so as to achieve quality control.

### Study status

The trial has been approved by the Ethics Committee (Gwll2022068) and was licensed in Meigu County and Yanyuan County of Liangshan Prefecture. The trial has also been registered in the ISRCTN registry (ISRCTN11299863). At the time of publication, we are preparing to recruit participants and collect the first round of baseline data, implemented as Protocol Version 1.0, dated June 2022. Any important protocol modifications will be communicated and recorded at the study home page https://www.isrctn.com/.

## Discussion

Improving health literacy is one of the most fundamental, economical and effective measures to improve health level of the whole people. Liangshan Prefecture declared comprehensive poverty alleviation at the end of 2020 [42], but the health literacy of residents in Liangshan Prefecture is still extremely low. Increasing the health literacy level of Liangshan Prefecture residents as soon as possible is an important measure to consolidate the achievement of poverty alleviation. Hence, we proposed a SW-CRT of health literacy intervention based on Family Branch System and Contracted Family Doctor Services when improving the traditional ICCC framework. The key technologies to be adopted include construction of electronic questionnaire quality control system, quality control based on artificial intelligence, mixed-effects model and causal inference. This study highlights family, using the unique Family Branch System and Contracted Family Doctor Services in Liangshan Prefecture to design intervention, and combines the mixed-effects model with CACE to estimate the intervention effect under non-compliance for the first time.

However, our study still has several limitations. First, there is a large migrant population in Liangshan Prefecture, so a relatively large loss to follow-up rate may exists. In view of this, we will not only expand the sample size during sample size calculation stage, but also give full play to the role of the sponsors of family branches to increase the publicity and education of study objects, so as to reduce the loss to follow-up. Second, due to the limitations of material and human resources in the actual work, we only carried this trial in two counties of Liangshan Prefecture. Nevertheless, this trial is only an exploration of the health literacy promotion program, and we may consider a larger health literacy intervention trial later.

In conclusion, this study explored an effective way to improve the health literacy of Yi nationality residents in Liangshan Prefecture, which can provide reference for other areas, especially poor areas, to improve residents’ health literacy.

## Supplementary Information

**Additional file 1: **CONSORT Extension for Cluster Trials 2012 Checklist.

**Additional file 2: **SPIRIT checklist.

## Data Availability

Not applicable.

## Abbreviations

Liangshan Prefecture: Liangshan Yi Autonomous Prefecture
FAMILY: Family-based Improvement for Health Literacy among the Yi nationality
NCDs: Noncommunicable diseases
CCM: Chronic Care Model
ICCC: Innovative Care for Chronic Conditions Framework
SW-CRT: Stepped wedge cluster randomised trial
CDC: Center for Disease Control and Prevention
CACE: Complier average causal effects
MCD: Minimum covariance determinant
